# MSBKA: A Multi-Strategy Improved Black-Winged Kite Algorithm for Feature Selection of Natural Disaster Tweets Classification

**DOI:** 10.3390/biomimetics10010041

**Published:** 2025-01-10

**Authors:** Guangyu Mu, Jiaxue Li, Zhanhui Liu, Jiaxiu Dai, Jiayi Qu, Xiurong Li

**Affiliations:** 1 School of Management Science and Information Engineering, Jilin University of Finance and Economics, Changchun 130117, China; guangyumu@jlufe.edu.cn (G.M.); 6221192039@s.jlufe.edu.cn (J.L.);; 2 Key Laboratory of Financial Technology of Jilin Province, Changchun 130117, China; 3 Changchun Community Official Staff College of Jilin Province, Changchun 130052, China; 4 Faculty of Information Technology, Beijing University of Technology, Beijing 100124, China

**Keywords:** black-winged kite algorithm, machine learning, social media platform, feature selection, natural disaster tweets, emergency response

## Abstract

With the advancement of the Internet, social media platforms have gradually become powerful in spreading crisis-related content. Identifying informative tweets associated with natural disasters is beneficial for the rescue operation. When faced with massive text data, choosing the pivotal features, reducing the calculation expense, and increasing the model classification performance is a significant challenge. Therefore, this study proposes a multi-strategy improved black-winged kite algorithm (MSBKA) for feature selection of natural disaster tweets classification based on the wrapper method’s principle. Firstly, BKA is improved by utilizing the enhanced Circle mapping, integrating the hierarchical reverse learning, and introducing the Nelder–Mead method. Then, MSBKA is combined with the excellent classifier SVM (RBF kernel function) to construct a hybrid model. Finally, the MSBKA-SVM model performs feature selection and tweet classification tasks. The empirical analysis of the data from four natural disasters shows that the proposed model has achieved an accuracy of 0.8822. Compared with GA, PSO, SSA, and BKA, the accuracy is increased by 4.34%, 2.13%, 2.94%, and 6.35%, respectively. This research proves that the MSBKA-SVM model can play a supporting role in reducing disaster risk.

## 1. Introduction

Natural disasters usually refer to events caused by abnormal changes in meteorology, geology, ocean, and ecology [1]. During the calamities, countless users publish posts on social media like Weibo and Twitter to communicate their status or seek assistance [2,3]. So, these platforms have become crucial data sources in emergency management [4]. Because of the complexity of natural disasters, decision-makers need to obtain vital information in the shortest possible time [5]. As shown in Table 1, informative tweets are closely related to disaster events and contribute to humanitarian relief [6], including infrastructure damage, casualties, and urgent demands. Unlike solely distinguishing whether tweets are relevant to the topic [7,8], the research on informative tweet recognition is more valuable for emergency response and situational awareness. In addition, technological progress makes it no longer difficult for researchers to obtain large-scale, real-time data from social media platforms [9]. Nevertheless, the speed of tweet generation is fast, and there is a severe problem of information overload [10]. Automatic and accurate classification of natural disaster tweets can assist the government and relevant departments in making efficient arrangements [11,12]. Therefore, this paper has specific practical significance.

As an elementary Natural Language Processing (NLP) task, text classification assigns data to predefined categories according to extracted features [13]. Identifying “Informative” or “Not Informative” tweets is a binary classification issue. Automatic text categorization must utilize classifiers [14], such as Naive Bayes (NB), Random Forest (RF), and Support Vector Machine (SVM). NB is conceived from the Bayes theorem and the independent hypothesis of characteristic conditions [15]. The category with the most significant probability is selected as the prediction result by calculating the conditional probability of each category under the given text features [16]. The NB model has a simple structure and high running efficiency. However, the hypothesis of feature independence is often not valid in reality, which will affect the classification outcome. RF is an integrated learning method based on the decision tree [17]. Each decision tree randomly opts for some data and features from the original data set for training. The final upshot is obtained by voting or averaging [18]. Due to the randomness, RF can decrease the risk of over-fitting and enhance the model’s generalization capability. Nevertheless, when the number of decision trees is large, the prediction process is slow, and more computational resources are needed. SVM follows the structural risk minimization principle and seeks the optimal hyperplane by maximizing the interval [19]. SVM is particularly able to solve the practical binary classification problem [20], but the kernel function must be used properly.

One of the prime challenges of text classification is that numerous irrelevant or redundant features impact the classifiers’ efficiency and accuracy [21]. Feature selection technology is specially adopted to deal with these difficulties. Choosing features with plentiful information and high correlation can enhance the model classification effect and reduce the computation cost [22,23]. In order to acquire ideal results, many scholars consider applying metaheuristic algorithms to feature selection, such as Genetic Algorithm (GA) [24,25], Particle Swarm Optimization (PSO) [26,27], and Sparrow Search Algorithm (SSA) [28,29]. The metaheuristic algorithm is exceedingly suitable for complicated optimization tasks. The principle is to discover the global optimal solution in the search space. BKA, proposed in 2024, simulates the behavior of black-winged kites in daily life and their formidable adaptability to environmental transformation [30]. With the merits of a straightforward framework, few manual parameters, and speedy convergence, BKA has been widely utilized in engineering [31,32,33]. Compared with other previous algorithms, BKA shows better competence. Nevertheless, no method is always reliable under the “No Free Lunch” theory [34]. The algorithm’s performance is a direct factor affecting feature selection. BKA’s global exploration and local exploitation are not balanced enough. The algorithm’s convergence accuracy is insufficient, and it is easy to fall into the local optimum. Therefore, making some corresponding improvements to BKA is necessary.

Here, we can obtain the following two motivations for this study:

(1) An applicable classifier must be chosen for the binary classification of natural disaster tweets.
(2) The improved metaheuristic algorithm can be used for feature selection to further increase the classification model’s accuracy.

This research’s primary contributions are as follows:

- We adopt three tactics to improve BKA. Firstly, we utilize the enhanced Circle mapping for population initialization. Secondly, we integrate the hierarchical reverse learning into BKA’s attack behavior. Thirdly, we introduce the Nelder–Mead method to obtain a better solution.
- We contrast the performance of several classification approaches and choose the SVM (Radial Basis Function, RBF kernel function) with the best result as the classifier. Then, we construct an MSBKA-SVM model based on the wrapper method’s principle.
- We conduct contradistinctive experiments with other metaheuristic algorithms on the tweet data set of four natural disasters. Empirical analysis demonstrates the proposed model’s superiority in feature selection and binary classification tasks.

## 2. Literature Review

### 2.1. Feature Selection

Feature selection is a data dimensionality reduction technology. According to the diverse interaction with the model training process, feature selection is split into the filter, embedding, and wrapper methods.

The filter method measures the features’ significance on the basis of statistical analysis, such as the Pearson Correlation Coefficient [35,36], Chi-square test [37,38], and Mutual Information [39,40]. This approach is carried out before model training and is independent of any specific algorithm [41]. Its main advantage is its ease of calculation, making it suited for large-scale data sets [42]. However, the filter method usually does not consider the features’ interaction and may ignore some essential features.

The embedding method allows the algorithm to decide which features to utilize. In other words, feature selection and model training are executed in simultaneity [43]. The embedding method is generally divided into two types: originating from penalty terms or tree models. The manners stemming from penalty terms include L1 Regularization (Lasso Regression) [44,45], L2 Regularization (Ridge Regression) [46,47], and Elastic-net Regression [48,49]. The approaches derived from the tree models contain Decision Tree [50,51], eXtreme Gradient Boosting (XGBoost) [52,53], and Light Gradient Boosting Machine (LightGBM) [54,55]. The embedding method is efficient, but it is difficult to determine a valid critical value to judge which features are momentous.

The wrapper method evaluates features based on the classifier’s performance [56], like accuracy or error. This approach regards the feature selection process as a search problem. Common means are Forward Selection [57,58], Backward Elimination [59,60], and Recursive Feature Elimination [61,62]. The wrapper method can consider the interaction and dependence between features [63]. It must try various feature subsets to train the model, so it is relatively accurate but more time-consuming.

In summary, the filter method ordinarily does not need complicated search strategies because of its independence from algorithms. The embedding method combines feature selection with the model training process, which commonly depends on the models’ internal mechanism. Metaheuristic algorithms are especially suitable for the wrapper method, given their high efficiency in dealing with complex optimization problems [64,65,66,67]. There are three prime reasons. Firstly, the wrapper method uses the model performance as the evaluation criterion of feature subsets. Metaheuristic algorithms can dynamically adjust tactics according to the feedback of model performance when searching for the optimal solution. The feature subsets with the most remarkable performance improvement are more likely to be found. Secondly, the wrapper approach contemplates the interaction between features and selects the optimal feature subsets by iteratively adding or removing features. Metaheuristic algorithms are able to handle this combinatorial optimization issue well. Thirdly, the wrapper method’s search space and calculation cost are comparatively large, so efficient algorithms are needed to reduce running expenses. Therefore, this research applies the metaheuristic algorithm to the feature selection on the basis of the wrapper method’s principle.

### 2.2. Classification of Natural Disaster Tweets

Social media platforms cover a variety of information [68,69], so scholars classify natural disaster tweets in diverse ways. The related research principally analyzes the content and sentiment expressed by tweets.

During calamities, data transmission and reception are normally mutual [70]. On the one hand, the affected people desire information about shelter, medical care, and food [71]. On the other hand, humanitarian organizations need to inform the current arrangements and provide the corresponding locations [72]. Identifying these specific contents in tweets promotes the rapid deployment of emergency response to natural disasters [73,74]. Related tweets are generally annotated as binary labels, such as urgent and not urgent [75] or high priority and low priority [76]. In addition, tweet data can be divided into fine-grained categories [77,78,79], including infrastructure damage, the trapped people’s situation, or the demand for relief materials.

Natural disasters’ unpredictability and severe destructiveness can easily induce negative emotions in netizens, leading to a public opinion crisis [80,81]. Identifying the sentiment tendencies assists relevant departments in evaluating the public’s views and attitudes toward emergencies [82,83]. Unlike lengthy articles or reports, tweets are limited by characters. This concise expression is conducive to understanding the textual emotions [84]. Sentiment polarities are classified into positive and negative [85] or positive, neutral, and negative [86]. Negative emotions ordinarily account for a more significant proportion in a particular period. Consequently, some sample synthesis techniques are applied to balance the data sets and ensure the model classification results’ accuracy [87]. Additionally, scholars usually concentrate on time and space to show the variational trend of emotional characteristics in diverse stages and regions [88,89,90].

Actually, only partial tweets are valuable for emergency management and situational awareness [91,92,93]. Tweets must first be filtered out of irrelevant text [94,95,96]. Then, the filtered data can be used to dig for beneficial content for disaster analysis [97]. However, being related to the incidents is not equivalent to contributing to humanitarian relief. This research is more specific and practical than merely distinguishing topic relevance.

## 3. Method

**Figure 1:**
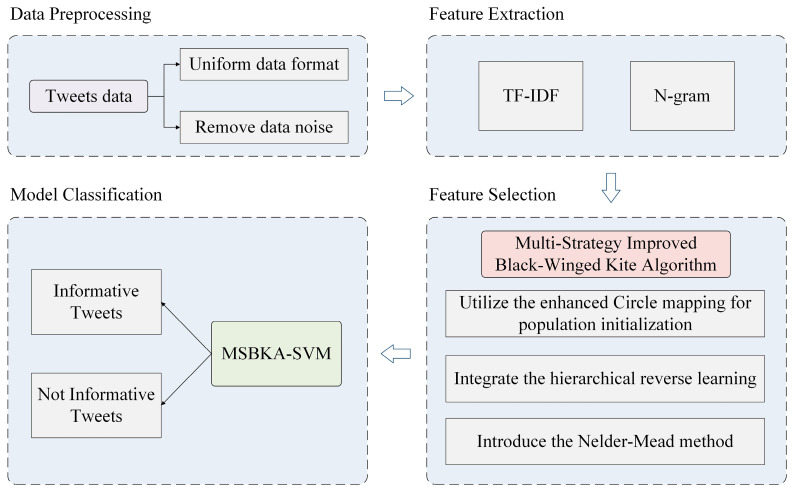
The architecture of the MSBKA-SVM model.

This study proposes an MSBKA for feature selection of natural disaster tweets classification. Figure 1 is the MSBKA-SVM model’s architecture, which comprises four modules: data preprocessing, feature extraction, feature selection, and model classification.

### 3.1. Data Preprocessing

Data preprocessing is the key to data analysis. The purpose is to improve data quality, uniform format, and remove noise. The specific steps are as follows:

- Remove URL links and special characters.
- Remove user names and topic tags.
- Remove punctuation and numbers.
- Convert all text contents to lowercase.
- Divide tweets into word lists.
- Filter out words less than three in length.
- Reconnect the remaining words into a string.

### 3.2. Feature Extraction

Unstructured text data cannot be immediately utilized for model training. This study adopts Term Frequency–Inverse Document Frequency (TF-IDF) combined with N-gram to convert tweets into numerical forms that classifiers can process. TF-IDF is a technique to measure the importance of words in document collections [98]. The TF-IDF value is directly proportional to the frequency of words emerging in a tweet but inversely proportional to the frequency of words appearing in the overall data set. The calculation process is shown in Equation (3).

(1) $${TF}_{i,\;j}=\frac{s_{i,\;j}}{\sum_{k}s_{k,\;j}}$$

(2) $${IDF}_{i}=\log\frac{S}{s_{i}}$$

(3) $$TF-IDF={TF}_{i,\;j}*{IDF}_{i}$$

where $s_{i,\;j}$ is the quantity of times a certain word emerges in the tweet j. $\sum_{k}s_{k,\;j}$ is the total quantity of words in the tweet j. S is the total quantity of tweets in the data set. $s_{i}$ is the quantity of tweets comprising a certain word.

N-gram selects n adjacent words from the text as features [99]. This study allows the model to consider single words (1-gram) and word pairs (2-gram) to capture more semantic information and contextual relationships. For the sake of improving the training efficiency and prediction performance, a scaling operation is performed to make the features all have an identical magnitude. Moreover, according to the ranking of TF-IDF values, up to 10,000 features are restricted for subsequent feature selection.

### 3.3. Black-Winged Kite Algorithm

A black-winged kite is a miniature bird with formidable hovering ability. The attack and migration behaviors inspire BKA. The algorithm’s population form is displayed in Matrix (4). The individual’s position is expressed as Equation (5). The parameters are described in Table 2.

(4) $$BK=\left[\begin{array}{ccccc} {\;BK}_{1,1} & {BK}_{1,2} & \cdots & \cdots & {\;BK}_{1,dim}\\ {\;BK}_{2,1} & {\;BK}_{2,2} & \cdots & \cdots & {\;BK}_{2,dim}\\ \vdots & \vdots & \vdots & \vdots & \vdots \\ \vdots & \vdots & \vdots & \vdots & \vdots \\ {\;BK}_{pop,1} & {\;BK}_{pop,2} & \cdots & \cdots & {\;BK}_{pop,dim\;} \end{array}\right]$$

(5) $${BK}_{i,\;j}={BK}_{lb}+rand\left({{BK}_{ub}-BK}_{lb}\right)$$

#### 3.3.1. The Attack Behavior

Black-winged kites feed on mammals and insects. During the flight, the bird will adjust the angle of its wings and tail in accordance with the wind speed, calmly hover to survey the prey, and then rapidly dive to onslaught. Two kinds of attack behaviors of BKA are indicated by Equation (6). The parameters are represented in Table 3.

(6) $$y_{t+1}^{i,\;j}=\left\{\begin{array}{c} y_{t}^{i,\;j}+n\left(1+\sin\left(r\right)\right)\times y_{t}^{i,\;j}\;p<r\\ y_{t}^{i,\;j}+n\left(2r-1\right)\times y_{t}^{i,\;j}\;else \end{array}\right.$$

(7) $$n=0.05\times e^{{-2\times \left(\frac{t}{T}\right)}^{2}}$$

#### 3.3.2. The Migration Behavior

In order to adapt to climate and environmental changes, black-winged kites migrate from north to south in winter. Leaders commonly lead bird migration. Their navigation capabilities are pivotal to the team’s achievement. BKA hypothesis: If the current individual’s fitness value $F_{i}$ is less than the random individual’s fitness value $F_{ri}$ in the population, the leader will abandon the leadership and enter the migration team. Instead, the leader will lead the population to their destinations. This dynamic selection strategy ensures the migration’s success, as Equation (8) expresses. The parameters are indicated in Table 4.

(8) $$y_{t+1}^{i,\;j}=\left\{\begin{array}{c} y_{t}^{i,\;j}+C\left(0,\;1\right)\times \left(y_{t}^{i,\;j}-L_{t}^{j}\right)\;F_{i}<F_{ri}\\ y_{t}^{i,\;j}+C\left(0,\;1\right)\times \left(L_{t}^{j}-m\times y_{t}^{i,\;j}\right)\;else \end{array}\right.$$

(9) $$m=2\times \sin\left(r+\pi /2\right)$$

The Cauchy distribution is a continuous probability distribution. The probability density function is shown in Equation (10).

(10) $$f\left(x\right)=\frac{1}{\pi \gamma \left[{1+\left(\frac{x-x_{0}}{\gamma }\right)}^{2}\right]},-\infty <x<\infty$$

When the scale parameter γ=1 and the location parameter $x_{0}=0$, the probability density function of the standard Cauchy distribution is indicated in Equation (11).

(11) $$f\left(x\right)=\frac{1}{\pi \left(x^{2}+1\right)},-\infty <x<\infty$$

### 3.4. Multi-Strategy Improved Black-Winged Kite Algorithm

Combining the Cauchy mutation and leader tactics enhances the original BKA’s search capability and convergence speed. Nevertheless, there is still a disequilibrium between global exploration and local exploitation. The algorithm has deficient optimization accuracy and easily falls into local optimization. Accordingly, MSBKA adopts three improved strategies.

#### 3.4.1. Utilize the Enhanced Circle Mapping for Population Initialization

**Figure 2:**
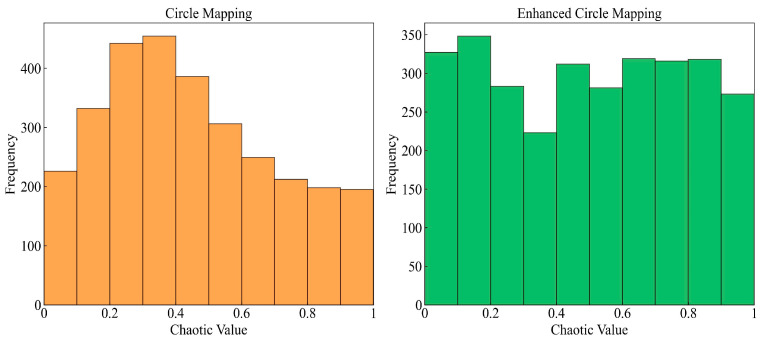
Frequency distribution histogram of initial solutions.

The incipient population’s diversity and quality are the pivotal factors affecting the metaheuristic algorithm’s performance [100]. The original BKA adopts a random way for population initialization, leading to the aggregation of generated individuals and low otherness. Chaotic mapping is ergodic and can explore all possible states in space [101]. The prime idea is to generate a chaotic sequence by mapping in the range [0, 1] and then convert the sequence into the population’s search space [102,103]. Among several types of chaotic mappings, Circle mapping is relatively steady and has higher coverage of chaotic values [104]. In order to avoid the chaotic values between [0.2, 0.4] densely, the traditional Equation (12) is improved to Equation (13) in this study. The initial solutions’ frequency distribution histogram is shown in Figure 2. The solutions’ dimension i is taken as 3000 to display the contrast effect intuitively. It is evident that the enhanced Circle mapping has more well-proportioned chaotic values.

(12) $$x_{i+1}=mod\left(x_{i}+0.2-\left(\frac{0.5}{2\pi }\right)\sin\left(2\pi x_{i}\right),1\right)$$

(13) $$x_{i+1}=mod\left(3.85\times x_{i}+0.4-\left(\frac{0.4}{3.85\pi }\right)\sin\left(3.85\pi x_{i}\right),1\right)$$

#### 3.4.2. Integrate the Hierarchical Reverse Learning

**Figure 3:**
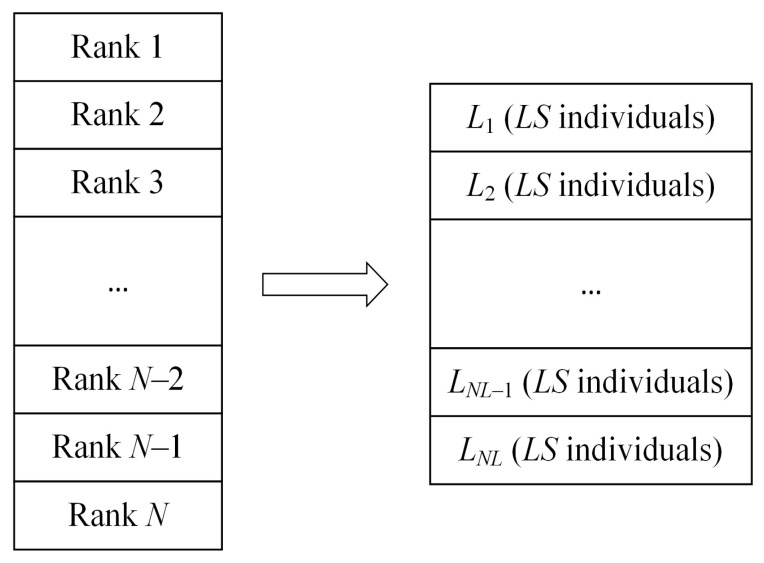
The schematic diagram of hierarchical reverse learning.

Individuals tend to gradually gather in some local areas in the iterative process, which makes the algorithm fall into local optimization [105,106]. In order to avoid premature convergence, MSBKA combines the original BKA’s attack behavior with the hierarchical reverse learning. This strategy needs to calculate each individual’s fitness value in the population and rank it as Rank 1, Rank 2, …, and Rank N from tiny to enormous. Then, N individuals are divided into NL levels, each with LS individuals. $L_{1}$ represents the individual’s best grade. $L_{NL}$ means the worst grade. The hierarchical schematic is indicated in Figure 3.

The reverse individual’s quality is not necessarily better than that of the current individual. There are still some individuals whose quality has declined after reverse learning. Accordingly, in this study, the obtained reverse individual $X_{opposite}$ and the previous level’s optimal individual $X_{j}^{best}$ are convexly combined to obtain the reverse individual tending towards excellence. This process can be expressed as Equation (14).

(14) $$X_{j}^{\prime }=\lambda {\;\times \;X}_{opposite}+\left(1-\lambda \right)\times X_{j}^{best}$$

#### 3.4.3. Introduce the Nelder–Mead Method

**Figure 4:**
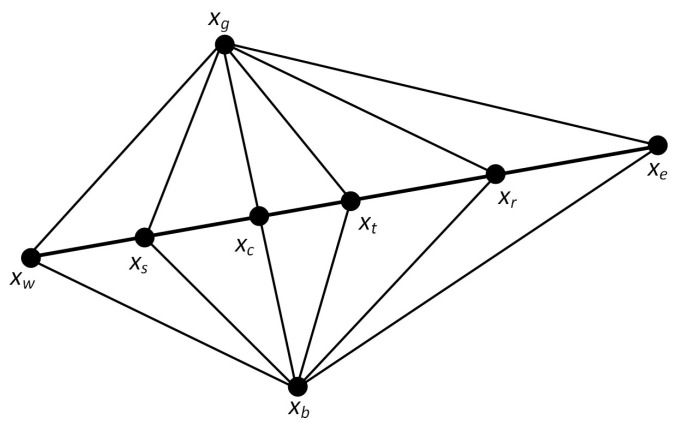
Search points of the Nelder–Mead method.

When faced with a complicated high-dimensional search space, the original BKA may have the issue of slow convergence or stagnation in some areas. Therefore, the Nelder–Mead method is introduced in this research. The polyhedron formed by connecting D+1 vertexes in D-dimensional space is called a simplex. The Nelder–Mead method is a simple and speedy local search algorithm [107]. The core idea is to obtain better points by performing several operations, replace the worst points, and approximate the best points by forming a novel polyhedron. MSBKA sets the Nelder–Mead method’s trigger condition as no improved fitness value every ten consecutive iterations. The Nelder–Mead method’s search points are shown in Figure 4.

The detailed operation steps are as follows:

(1) Calculate the fitness values of all vertexes and seek out the best point $x_{g}$, the second-best point $x_{b}$, and the worst point $x_{w}$. The fitness values are $f\left(x_{g}\right)$, $f\left(x_{b}\right)$, and $f\left(x_{w}\right)$. Calculate the center point’s position by Equation (15).
  (15) $$x_{c}=\frac{x_{g}+x_{b}}{2}$$
(2) The reflection operation is performed by Equation (16). Reflect the worst point $x_{w}$ according to the center point $x_{c}$. α is the reflection coefficient. The reflection point’s fitness value is $f\left(x_{r}\right)$.
  (16) $$x_{r}=x_{c}+\alpha \left(x_{c}-x_{w}\right)$$
(3) When $f\left(x_{r}\right)>f\left(x_{g}\right)$, the reflection direction is correct. Then, the extension operation is executed by Equation (17). μ is the extension coefficient. The extension point’s fitness value is $f\left(x_{e}\right)$. When $f\left(x_{e}\right)>f\left(x_{g}\right)$, displace the worst point $x_{w}$ with the extension point $x_{e}$. Otherwise, displace the worst point $x_{w}$ with the reflection point $x_{r}$.
  (17) $$x_{e}=x_{c}+\mu \left(x_{r}-x_{c}\right)$$
(4) When $f\left(x_{r}\right)<f\left(x_{g}\right)$, the reflection direction is false. Then, the compression operation is performed by Equation (18). β is the compression coefficient. The compression point’s fitness value is $f\left(x_{t}\right)$. When $f\left(x_{t}\right)>f\left(x_{w}\right)$, replace the worst point $x_{w}$ with the compression point $x_{t}$.
  (18) $$x_{t}=x_{c}+\beta \left(x_{w}-x_{c}\right)$$
(5) When $f\left(x_{w}\right)<f\left(x_{r}\right)<f\left(x_{g}\right)$, the shrinkage operation is executed by Equation (19). δ is the shrinkage coefficient. The shrinkage point’s fitness value is $f\left(x_{s}\right)$. When $f\left(x_{s}\right)>f\left(x_{w}\right)$, displace the worst point $x_{w}$ with the shrinkage point $x_{s}$. Otherwise, displace the worst point $x_{w}$ with the reflection point $x_{r}$.
  (19) $$x_{s}=x_{c}-\delta \left(x_{w}-x_{c}\right)$$

#### 3.4.4. The Running Steps of MSBKA

The MSBKA’s running steps are as follows:

**Step 1**: Set the related parameters of MSBKA.

**Step 2**: Initialize the population using the enhanced Circle mapping by Equation (13).

**Step 3**: Evaluate the fitness value of each individual. The individual with the best fitness value is determined as the leader in the initial population.

**Step 4**: If a random number r within [0, 1) is less than 0.4, the attack behavior is executed by Equation (6). Otherwise, the migration behavior is performed by Equation (8). In the attack behavior, the hierarchical reverse learning strategy is carried out by Equation (14) with a probability of 0.5.

**Step 5**: Update the current optimal solution and fitness value. Determine whether to perform the Nelder–Mead method utilizing Equations (15)–(19).

**Step 6**: Repeat Steps 4 to 5. Output the global optimal solution and fitness value when reaching the maximum quantity of iterations.

#### 3.4.5. The Time Complexity of MSBKA

Time complexity is utilized to evaluate the algorithm’s efficiency, which reflects the relationship between the time required for algorithm execution and input data size. Big O notation is a standard method [108]. This approach describes the algorithm’s performance in the worst condition. Set the maximum iterations T, the population scale N, and the problem dimension D. The BKA’s time complexity is represented as $O\left(N\times D\times T\right)$. The influence of three improved strategies adopted by MSBKA on the time complexity is limited. Chaotic initialization does not directly participate in the subsequent iterative process. The extra calculation involved in reverse learning is linear for the individuals. The Nelder–Mead method has a trigger condition. Therefore, MSBKA has the same order of magnitude in time complexity as the original BKA.

### 3.5. Feature Selection

**Figure 5:**
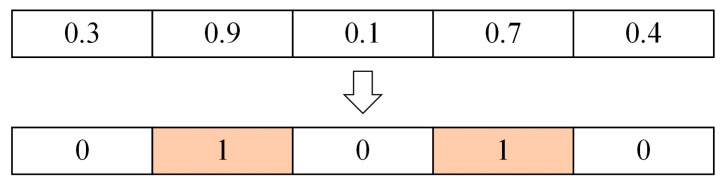
Schematic diagram of the feature selection process.

The essence of feature selection is to let individuals search in the space composed of the data set’s feature dimensions. The metaheuristic algorithm’s individual represents the potential solution. Each iteration’s update of individual positions equals the feature subsets’ renewal. The individual with the highest fitness value is the global optimal solution. The corresponding feature subset is considered the optimal feature subset. If the data set has n features, then each individual is a vector of length n. In the initial population generated by MSBKA, each position in the vector is given a random value within [0, 1]. When this value exceeds 0.5, it is set to 1, indicating that the feature is selected. If this value is less than or equal to 0.5, it is set to 0, signifying that the feature is not selected. As shown in Figure 5, assuming that the data set contains five features, only the second and fourth features in the current individual are selected.

The feature selection is aimed at seeking out the feature subset with the highest accuracy and the least quantity of features. Consequently, the fitness function is defined as Equation (20).

(20) $$Fitness=\omega \times Accuracy-\left(1-\omega \right)\times \frac{R}{C}$$

R is the number of selected features. C is the total quantity of features in the data set.

### 3.6. Model Classification

This study utilizes several models to implement the binary classification of tweets, comprising Naive Bayes, Random Forest, and Support Vector Machine. Among them, SVM selects Linear and RBF kernel functions. These two expressions are expressed as Equations (21) and (22).

(21) $$K\left(x,\;y\right)=\left(x\cdot y\right)$$

(22) $$K\left(x,\;y\right)=e^{\left(\frac{-{\left\|x-y\right\|}^{2}}{2\sigma^{2}}\right)}$$

## 4. Empirical Analysis

### 4.1. Data Acquisition

CrisisMMD is a Twitter data set about natural disasters [109,110]. Each tweet is marked as “Informative” or “Not Informative”, with labels 1 and 0, respectively. This study selects four types of natural disasters: Sri Lanka Floods, Mexico Earthquake, Hurricane Maria, and California Wildfires. Finally, 4370 tweets are retained after filtering and duplicate items processing. The number of labels in the two categories is balanced at 2185. The descriptions of four natural disaster events are indicated in Table 5.

### 4.2. Experimental Details

The training set and the test set are randomly selected in a ratio of eight to two. The other parameter settings are indicated in Table 6.

### 4.3. Evaluation Metrics

For the purpose of comprehensively contrasting diverse classification models, this research adopts four evaluation metrics: *accuracy*, *precision*, *recall*, and *F*1. The normative binary classification confusion matrix is indicated in Table 7. The higher the values of all evaluation metrics, the better the performance of the classifiers. The calculation processes are expressed by Equations (23)–(26).

(23) $$Accuracy=\frac{TP+TN}{TP+FP+FN+TN}$$

(24) $$Precision=\frac{TP}{TP+FP}$$

(25) $$Recall=\frac{TP}{TP+FN}$$

(26) $$F1=\frac{2*Recall*Precision}{Precision+Recall}$$

### 4.4. Experimental Results

#### 4.4.1. The Selection of Classifiers

Before combining with the metaheuristic algorithms, this research contrasts Naive Bayes, Random Forest, and SVM with two kernel functions. As shown in Table 8, SVM with RBF kernel function has the best performance. The accuracy reaches 0.7426. Compared with Naive Bayes, Random Forest, and SVM with a linear kernel function, the accuracy is improved by 4.68%, 2.05%, and 3.18%, respectively. Consequently, this study selects SVM with RBF kernel function as the feature selection task’s classifier.

#### 4.4.2. The Evaluation Metrics Comparison of Classification Models

This study compares other metaheuristic algorithms to attest to the proposed model’s advantage in tweet classification, including GA, PSO, SSA, and BKA. The whole experiment is conducted in identical conditions and parameter settings to guarantee the credibility of the contrast results. The evaluation metrics are indicated in Table 9. The separate evaluation of two data labels can analyze the model performance in more detail.

**Figure 6:**
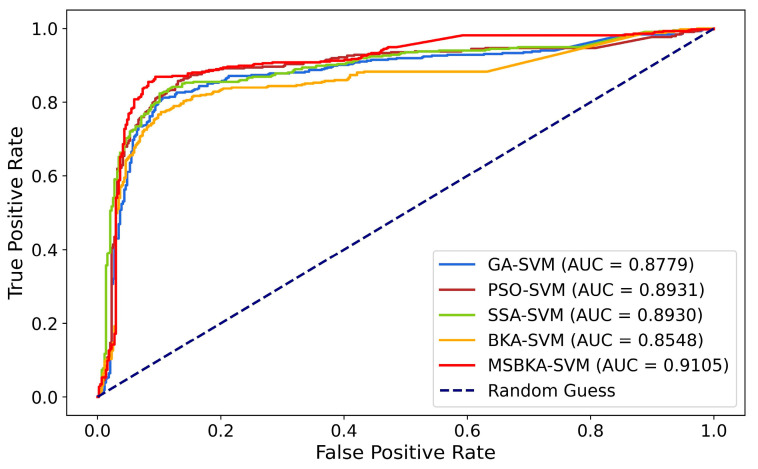
The ROC-AUC diagram of five classification models.

The Receiver Operating Characteristic (ROC) curve and the Area Under the ROC Curve (AUC) are vital indexes for measuring the binary classification model’s performance. ROC is realized by plotting the relationship between the True Positive Rate (*TPR*) and False Positive Rate (*FPR*). The range of AUC is from 0.5 (random guess) to 1 (perfect classifier), which quantifies the model’s capability to distinguish diverse categories. Figure 6 shows the ROC-AUC diagram of five classification models.

(27) $$TPR=\frac{TP}{TP+FN}$$

(28) $$FPR=\frac{FP}{FP+TN}$$

The evaluation metrics of five classification models reveal the following findings:

- The classifier’s accuracy is strikingly increased after feature selection combined with the metaheuristic algorithms. Compared with individual SVM (RBF kernel function), the accuracy of GA-SVM, PSO-SVM, SSA-SVM, BKA-SVM, and MSBKA-SVM is improved by 13.86%, 16.32%, 15.41%, 11.70%, and 18.80%, respectively.
- By utilizing the enhanced Circle mapping, integrating the hierarchical reverse learning, and introducing the Nelder–Mead method, MSBKA’s performance is better than that of the original BKA. Furthermore, MSBKA shows the most remarkable enhancement among all the models. Its accuracy achieves 0.8822, heightened by 4.34%, 2.13%, 2.94%, and 6.35% compared to GA, PSO, SSA, and BKA. The main reason is that these original algorithms have some defects, which lead to poor stability.
- MSBKA-SVM has the highest AUC value of 0.9105, which is 3.71%, 1.95%, 1.96%, and 6.52% higher than GA-SVM, PSO-SVM, SSA-SVM, and BKA-SVM. The proposed model’s AUC is the closest to 1. In other words, the capability to distinguish two categories is excellent.

#### 4.4.3. The Performance Contrast of Classification Models

**Figure 7:**
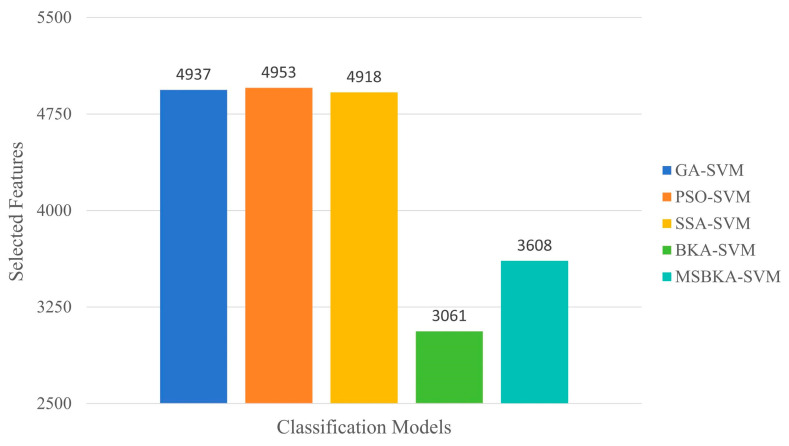
The number comparison of features selected by five classification models.

**Figure 8:**
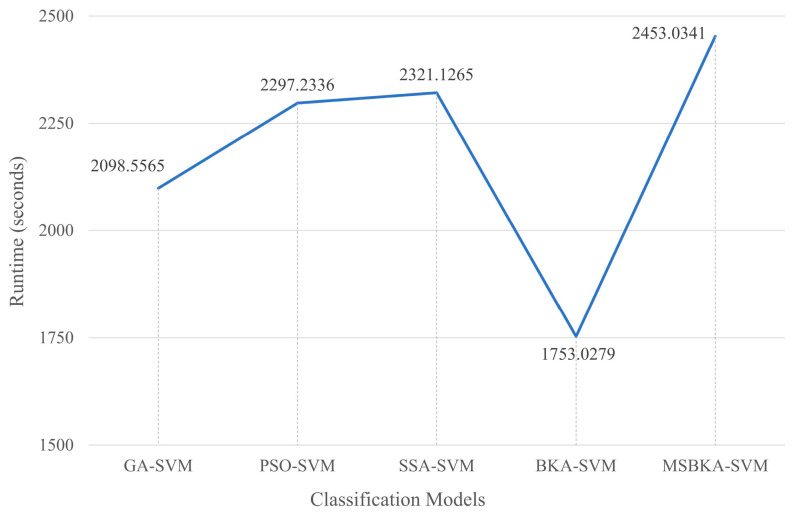
The runtime comparison of five classification models.

**Figure 9:**
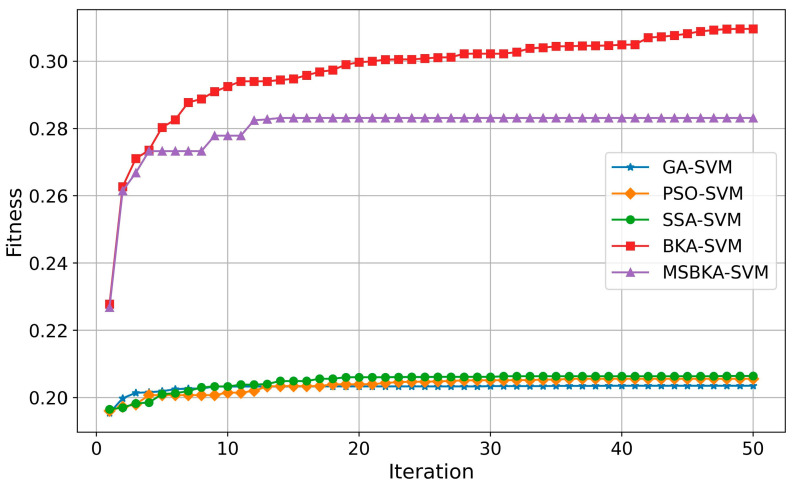
The convergence curves of five classification models.

In addition to accuracy, the quantity of features selected by classification models and the running time also need to be considered. Furthermore, to show the optimization algorithm’s performance changes in the iterative process, the fitness value is utilized as an evaluation criterion to plot the convergence curve. The comparison results of these aspects are indicated in Figure 7, Figure 8 and Figure 9.

As shown in Figure 7, in contrast with GA-SVM, PSO-SVM, SSA-SVM, and MSBKA-SVM, the number of selected features for BKA-SVM is minimal. Nevertheless, the accuracy of BKA-SVM is not high enough. This phenomenon is due to the features selected by BKA-SVM that are irrelevant or not helpful to model classification. The quantity of features selected by MSBKA-SVM is slightly more than that of BKA-SVM, but the accuracy is dramatically improved. That is to say, MSBKA-SVM can select the most relevant and critical features from text data. Similarly, Figure 8 shows that BKA-SVM has the minimum running time and MSBKA-SVM has the maximum execution time among all classification models. In Figure 9, the fitness function set in this study takes into account both the classification accuracy and the number of selected features. The fitness value of MSBKA-SVM is lower than that of BKA-SVM. However, this research focuses on practical applications, and accuracy is obviously the most pivotal criterion. In summary, combined with various factors, the proposed model is still optimal.

## 5. Conclusions and Prospect

### 5.1. Conclusions

Identifying informative tweets related to natural disasters avails disaster relief assignments. The irrelevant and redundant features in tweets will affect the classification accuracy. These issues can be resolved by using feature selection technology. A novel metaheuristic algorithm, BKA, is suitable for complicated optimization tasks. However, BKA suffers from inadequate search accuracy and easily falls into the local optimum. There is still room for capability advancement.

This study proposes a multi-strategy improved black-winged kite algorithm for feature selection of natural disaster tweets classification. Specifically, three optimization tactics are adopted to handle the BKA’s deficiencies. Firstly, the enhanced Circle mapping is utilized for population initialization. Secondly, hierarchical reverse learning is integrated into BKA’s attack behavior. Thirdly, the Nelder–Mead method is introduced to obtain a better solution. Among all classifiers, SVM (RBF kernel function) performs best. The hybrid MSBKA-SVM model based on the wrapper method’s principle sufficiently combines the metaheuristic algorithm and machine learning.

Experimental results display that feature selection plays a positive role in model classification. Compared with individual SVM (RBF kernel function), the accuracy of GA-SVM, PSO-SVM, SSA-SVM, BKA-SVM, and MSBKA-SVM is improved by 13.86%, 16.32%, 15.41%, 11.70%, and 18.80%, respectively. Furthermore, MSBKA shows the most conspicuous enhancement among all the models. Its accuracy achieves 0.8822, increased by 4.34%, 2.13%, 2.94%, and 6.35% compared to GA, PSO, SSA, and BKA. Meanwhile, considering the quantity of selected features, the model runtime, and the fitness value, the proposed model is still the most ideal scheme.

On the whole, this research has prominent pragmatic implications. It is challenging to discriminate complex tweet content. The MSBKA-SVM’s classification capability is more distinguished than that of other methods. Contrast experiments demonstrate the proposed model’s advantage in dealing with natural disaster tweets. Accordingly, the government and relevant departments can immediately handle the identified vital information to reduce the damages caused by calamities and prevent a large-scale public opinion crisis.

### 5.2. Limitation and Future Direction

This research still has certain limitations. Due to the Internet’s anonymity, there is much false or unverified information on social media platforms. Especially during emergencies, these forged contents can easily cause public panic and social unrest, even threatening national security. Identifying informative tweets about natural disasters is merely the first phase of emergency management. Organizing and utilizing these tweets effectively and detecting false information simultaneously is an issue that needs further contemplation.

## Figures and Tables

**Table 1:** The instances of tweets with two labels.

Tweets	Labels
We visited Puerto Rico today!	Not Informative
Ten nurses and one doctor from @UR_Med are leaving this AM to help in Puerto Rico. Hear from them on @13WHAM.	Informative

**Table 2:** The elaborations of the parameters in Equations (4) and (5).

Parameters	Elaborations
pop	The population size
dim	The optimization problem’s dimension
lb	The lower boundary
ub	The upper boundary
rand	A random value within the interval [0, 1]

**Table 3:** The elaborations of the parameters in Equations (6) and (7).

Parameters	Elaborations
$y_{t}^{i,\;j}$, $y_{t+1}^{i,\;j}$	The ith black-winged kite’s position information in the jth dimension at the tth and t+1th iterations
r	A random digit from 0 to 1
p	A constant of 0.9
n	A dynamically adjusted variable
t	The current iteration quantity
T	The maximum quantity of iterations

**Table 4:** The elaborations of the parameters in Equations (8) and (9).

Parameters	Elaborations
$L_{t}^{j}$	The leader’s location in the jth dimension at the tth iteration
$C\left(0,\;1\right)$	The Cauchy distribution function
m	A dynamically adjusted variable

**Table 5:** Descriptions of four natural disaster events.

Natural Disaster Events	Start Date	End Date	Tweet Counts
Sri Lanka Floods	31 May 2017	3 July 2017	460
Mexico Earthquake	20 September 2017	6 October 2017	580
Hurricane Maria	20 September 2017	13 November 2017	2770
California Wildfires	10 October 2017	27 October 2017	560

**Table 6:** The parameter settings of the whole experiment.

Parameters	Value
T	50
N	50
Chaotic iteration	150
λ	0.5
α	1
μ	2
β	0.5
δ	0.5
ω	0.5

**Table 7:** The normative binary classification confusion matrix.

	Predicted Positive Example	Predicted Negative Example
**Factual Positive Example**	True Positive (*TP*)	False Negative (*FN*)
**Factual Negative Example**	False Positive (*FP*)	True Negative (*TN*)

**Table 8:** The evaluation metrics contrast of several classifiers.

Classifiers	*Accuracy*	Labels	*Precision*	*Recall*	*F*1
Naive Bayes	0.7094	0	0.7798	0.5835	0.6675
		1	0.6673	0.8352	0.7419
Random Forest	0.7277	0	0.6970	0.8055	0.7473
		1	0.7696	0.6499	0.7047
SVM (Linear)	0.7197	0	0.7388	0.6796	0.7080
		1	0.7034	0.7597	0.7305
SVM (RBF)	0.7426	0	0.7409	0.7460	0.7434
		1	0.7442	0.7391	0.7417

**Table 9:** The evaluation metrics comparison of five classification models.

Models	*Accuracy*	Labels	*Precision*	*Recall*	*F*1
GA-SVM	0.8455	0	0.8268	0.8741	0.8498
		1	0.8665	0.8169	0.8410
PSO-SVM	0.8638	0	0.8597	0.8696	0.8646
		1	0.8681	0.8581	0.8631
SSA-SVM	0.8570	0	0.8467	0.8719	0.8591
		1	0.8679	0.8421	0.8548
BKA-SVM	0.8295	0	0.7869	0.9039	0.8413
		1	0.8871	0.7551	0.8158
MSBKA-SVM (Proposed)	0.8822	0	0.8568	0.9176	0.8862
		1	0.9113	0.8467	0.8778

## Data Availability

The raw data and code supporting the conclusions of this article will be made available by the authors on request.
